# Provider and client perspectives on the use of maternity waiting homes in rural Rwanda

**DOI:** 10.1080/16549716.2023.2210881

**Published:** 2023-05-16

**Authors:** Edwin Tayebwa, Samwel Maina Gatimu, Richard Kalisa, Young-Mi Kim, Jeroen van Dillen, Jelle Stekelenburg

**Affiliations:** a Department of Health Sciences, Global Health, University Medical Centre Groningen, Groningen, The Netherlands; b Research Department, Diabetes Foot Foundation of Kenya, Nairobi, Kenya; c School of Public Health, University of Rwanda, Kigali, Rwanda; d Jhpiego, Johns Hopkins University, Baltimore, MD, USA; e Amalia Children’s Hospital, Radboudumc Nijmegen, Nijmegen, The Netherlands; f Department of Obstetrics and Gynaecology, Leeuwarden Medical Centre, Leeuwarden, The Netherlands

**Keywords:** Perceptions, facilitators, barriers, maternity waiting home use, provider and client perspectives

## Abstract

**Background:**

The World Health Organization recommends the implementation of maternity waiting homes (MWH) to reduce delays in access to obstetric care, particularly for high-risk pregnancies and mothers living far from health facilities, and as a result, several countries have rolled out MWHs. However, Rwanda has not implemented this recommendation on a large scale. There is only one MWH in the country, hence a gap in knowledge regarding the potential utilisation and benefits of MWHs.

**Objective:**

To explore providers’ and clients’ perspectives on facilitators and barriers to the use of MWH in rural Rwanda.

**Methods:**

We conducted a qualitative study to explore health providers’ and clients’ perspectives on facilitators and barriers to the use of MWH in Rwanda, between December 2020 and January 2021. We used key informant interviews and focus group discussions to collect data. Data were analysed using NVivo qualitative analysis software version 11.

**Results:**

Facilitators included perceptions that the MWH offered either a peaceful and home-like environment, good-quality services, or timely obstetric services, and was associated with good maternal and neonatal outcomes. Barriers included limited awareness of the MWH among pregnant women, fear of health providers to operate the MWH at full capacity, women’s lack of autonomy, uncertainty over funding for the MWH, and perceived high user fees.

**Conclusion:**

The Ruli MWH offers a peaceful environment for pregnant women while providing quality and timely obstetric care, resulting in positive maternal and neonatal outcomes for women. However, its existence and benefits are not widely known, and its use is limited due to inadequate resources. There is a need for increased awareness of the MWH among healthcare providers and the community, and lessons from this MWH could inform the scale up of MWHs in Rwanda.

## Introduction

Despite a 79% decline in the maternal mortality ratio from 1,071 deaths per 100,000 live births in 2000 to 203 in 2020 [1–3], maternal deaths remain a challenge in Rwanda [4], with many women still dying from preventable causes. Postpartum haemorrhage, hypertensive disorders, abortion, and obstructed labour, which are mainly attributed to delays in seeking health services, reaching care, and receiving adequate healthcare, are the major causes of maternal deaths [5–7].

Several interventions have been implemented in Rwanda to reduce maternal morbidity and mortality [8]. The maternal mortality ratio has, however, plateaued over the last 5 years [4], necessitating a review of these approaches and whether any other approaches could be implemented to accelerate the reduction of maternal mortality in Rwanda. One such approach is the implementation of maternity waiting homes (MWH), which is recommended by the World Health Organization as an intervention to contribute to reducing delays in access to obstetric care [9]. An MWH is a residential structure, built near a health facility offering obstetric services, to provide accommodation for pregnant women nearing the end of their pregnancy or post-delivery [10]. While earlier studies lacked evidence on the benefits of MWHs [11], the more recent reviews and meta-analyses have found that MWHs increased access to skilled birth attendance [12], reduced maternal mortality [13], increased postnatal visits [12], improved access to maternity care [12,14], and demonstrated a protective effect on maternal mortality in low-resource settings [15]. Similarly, in Rwanda, MWH users had fewer caesarean deliveries, stillbirths, and postpartum haemorrhage cases compared to MWH non-users [16].

MWHs have been implemented in many countries, but with varying approaches [9,11,12]. In some settings, MWHs have been built in the communities, while in others, they have been built near health facilities providing basic emergency obstetric and neonatal care (EmONC) or comprehensive EmONC [17] However, studies have shown that the utilisation of MWHs is associated with socio-economic, demographic, socio-cultural, and facility-related factors [18–21]. Among the user-related constraints include awareness of pregnancy-related complications [19], a lack of geographical access and knowledge about the existence of MWHs [20], a lack of husbands’ support [19], women’s lack of decision-making autonomy [21], perceived quality of care [19], and low socio-economic status [21]. Facility-related factors include the provision of culturally inappropriate care [20], lack of funding [20], health service integration, and lack of provision of basic healthcare needs while in the MWHs [21]. In Rwanda, MWHs are not prioritised among the key interventions being implemented to reduce maternal mortality and only one MWH exists. Factors influencing the use of this MWH in Rwanda have not been studied, and, therefore, an understanding of socio-cultural and structural influences on the use and non-use of MWHs from beneficiaries’ and health providers’ perspectives is critical in shaping the future of MWH in Rwanda. Hence, we explored the providers’ and clients’ perspectives on facilitators and barriers to the use of MWH in rural Rwanda.

## Methods

### Study design and setting

We conducted a qualitative study among clients and health providers at the MWH located at Ruli Hospital in the mountainous Gakenke District in the Northern Province of Rwanda. Ruli Hospital is a district-level hospital that serves a catchment population of about 110,548 inhabitants (projections from the 2012 census) from within the Gakenke District and beyond [22]. Just like elsewhere in Rwanda, health centres in the district are run by nurses and midwives who provide routine maternal and child health services, including basic emergency obstetric and newborn care. Whenever health centres receive mothers with obstetric complications, they refer them to district hospitals for comprehensive EmONC services, delivered by more experienced midwives and medical doctors. Some health centres in the district identify women with high-risk pregnancies and refer them to the MWH to facilitate their timely access to quality obstetric care. At the hospital, the women are received by more experienced midwives and medical doctors who, in turn, may decide to admit the women to the MWH based on the following criteria: a problem related to pregnancy, e.g. history of abortion; a caesarean section; prolonged labour, as well as problems during the current pregnancy, including preterm premature rupture of membranes, antepartum haemorrhage, reduced foetal movement, pre-eclampsia, etc., and one of the following conditions: at least 36 weeks of amenorrhoea, residing far from the hospital (more than 3 h of walking), woman having no caretaker at home, victims of gender-based violence, low socio-economic status, and clinicians’ decision. Upon their admission to the MWH, pregnant women receive obstetric care, psycho-social care, education on proper nutrition, breastfeeding, and birth preparedness. The social workers at the MWH facilitate the women to gain hands-on skills such as gardening, cooking, making handicrafts, and knitting baby items.

### Sampling and recruitment procedures

We utilised purposeful sampling to enrol pregnant women who were in the MWH (users), postpartum mothers who did not use the MWH, and health care providers at the hospital. All MWH users were pregnant women who were residing at the MWH at the time of data collection. All MWH non-users were mothers who had delivered and were admitted to the postpartum ward. The MWH users and non-users had similar characteristics, and both groups qualified for admission to the MWH. Health providers were identified and found at their places of work, and they included nurses, midwives, nutritionists, social workers, administrators, and a private donor. The MWH users and non-users participated through four focus group discussions (FGDs), with the minimum number of participants being 6 and the maximum being 8, while health providers participated through eight individual key informant interviews (KIIs). All FGDs and KIIs were conducted within Ruli Hospital at locations that were deemed convenient by the respondents, with only the interviewers and respondents being present during the interviews.

### Data collection

Data were collected in December 2020 and January 2021 by the first and third authors with support from one experienced qualitative research associate using pretested open-ended FGDs and KIIs guides which were developed in English and translated into Kinyarwanda. All the data collectors were maternal and child health experts in low-resource settings. The interviews were conducted in Kinyarwanda. In FGDs, we encouraged participants to contribute freely, and each session took approximately 60–90 min. At the beginning of the FGDs and KIIs, interviewers briefed participants on the study aims and informed them that the conversation would be audio-recorded and verbal consent obtained before the start of the interview. Data collection was stopped when a similar pattern of responses was established, and no additional information was obtained from further interviews.

### Data analysis

The data were transcribed verbatim in Kinyarwanda and then translated into English by the first author and a research assistant to increase their familiarity with the data. The transcripts were then entered into NVivo qualitative analysis software version 11 (QSR International, Victoria, Australia), and inductive thematic analysis was conducted [23]. The first three authors independently read and re-read the transcripts and coded them line by line to identify meaning units. Similar meaning units were grouped into condensed meaning units and then coded to expound on the condensed meaning units before grouping them through an inductive and deductive process to develop categories. Consensus on the meaning units and themes was reached through discussions, first among the three first authors and later validated by all authors. Categories were verified with the data to ensure they reflected the content of the transcripts, and constant comparison was made between categories to develop final themes.

### Ethical considerations

The study was approved by the Rwanda National Ethical Committee, the Rwanda National Health Research Committee, and the Rwanda Biomedical Centre. All participants provided written informed consent and voluntarily participated in the study. Personal identifiers were not recorded.

### Results

**Figure 1.**
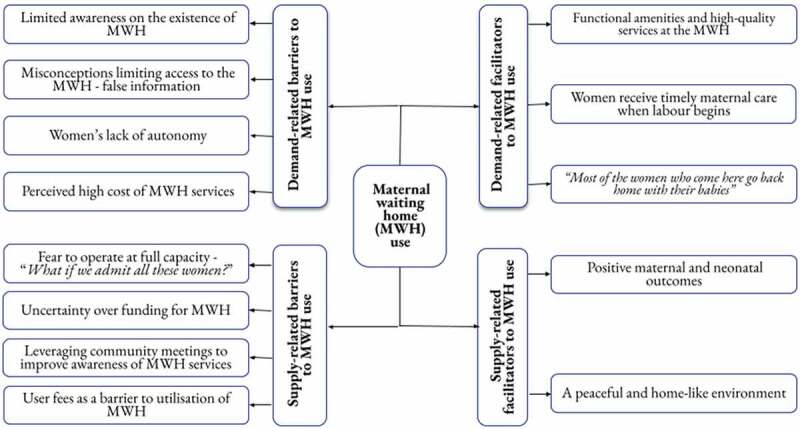
Respondents’ perspectives on barriers and facilitators to MWH use.

During the data analysis, four categories emerged from the FGDs and KIIs, related to MWHs, which we labelled ‘demand-supply–related barriers’ and ‘demand-supply–related facilitators’, with each subdivided into several subcategories. Respondents’ perspectives are summarised as shown in Figure 1.

## Facilitators of MWH use

### Demand-related facilitators

#### Functional amenities and high-quality services at the MWH

Most participants reported that the MWH services provided at Ruli Hospital catered for their social, psychological, and financial needs. The MWH maintains a high level of cleanliness and hygiene with fully equipped private rooms, private showers, a kitchen with necessary utensils, and a store to keep fresh food. Mothers prepare meals based on their choices:

… This MWH is clean, has a big store to keep our raw food, and has an area to change clothes or do personal stuff when there is more than one woman at a time. (FGD 2, MWH user).

#### Women receive timely maternity care when labour begins

The MWH allows pregnant women to get closer to skilled care before labour starts. Most participants stated that women in the MWH benefit from timely delivery services when labour begins, including being referred to a referral/tertiary hospital on time if additional care is required. An MWH non-user said that staying at the MWH offers an opportunity to avoid delivery by traditional birth attendants in the communities.

[…] one of the most important reasons for deciding to stay at the waiting home is to get timely midwifery care […]. … staying at the MWH is a good opportunity to avoid care from traditional birth attendants (MWH non-user, FGD 1).

#### ‘Most of the women who come here go back home with their babies’

Most MWH users reported that the MWH has contributed to good maternal and neonatal outcomes for most beneficiaries and has a good reputation among pregnant women. As a result, most women who stay at the MWH deliver well and go back home with their babies.

I knew a neighbour who was attended to in this MWH in 2018, she used to have recurring abortions. She told me that she was admitted to this MWH and healthcare providers took care of her until she delivered. Based on that testimony when the doctor told me that I was going to be admitted to our MWH, I never hesitated *…* (MWH user, FGD 2).

### Supply-related facilitators

#### A peaceful and home-like environment

Both MWH users and health providers noted that the MWH is a safe environment where quality maternal care services are provided. They also noted that the MWH provided a homely environment where users remain in contact with their families and friends during their stay:

Expectant mothers are safe; they talk with each other and are well-taken care of. More importantly, they are still in contact with their families. (Ruli Hospital, KII 4).

There was consensus among participants that quality services have resulted in a strong brand and good reputation among mothers who use it. Because such positive stories move across communities and districts, the MWH receives women from all over the Gakenke District and from several neighbouring districts: ‘

… it is even good for our hospital branding as it is the first one in the country, … people in this community appreciate the MWH as they have heard good things about it. (Ruli Hospital, KII 1).

#### Positive maternal and neonatal outcomes

There was consensus among key informants (health providers) that maternal health outcomes for high-risk mothers have improved among MWH users. One of the key informants noted that no maternal death had occurred among MWH users in the past 5 years:

Absolutely, I have not heard any story of a woman who died while in the MWH … (Ruli Hospital, KII 2).

## Barriers to MWH use

### Demand-related barriers

#### Limited awareness of the existence of the MWH

Some participants among MWH users and non-users reported knowing about MWH activities after being informed by a doctor or midwife when they came to the maternity ward. The MWH non-users said that healthcare workers at the antenatal care clinics they attended in health centres did not inform them about the availability of MWH services. They noted that health centre staff could play a key role in raising awareness of the existence, and importance, of the MWH, thereby increasing its benefits and utilisation among eligible women in the region.

*…* Nobody has ever informed me about an MWH in all different seven health centres I had attended my antenatal care. … , I believe it’s important, especially for women who want to come here [MWH] particularly those who live far away (MWH non-user, FGD 1).

#### Women’s lack of autonomy

Most participants reported a lack of autonomy in the decision-making process for them to stay at the MWH. They said that husbands usually determine whether, or not, their wives stay at the MWH and that in some cases, mothers-in-law were also involved in the decision-making process. Most women who stayed at the MWH said that their husbands were the ones who chose to let them stay there.

We came here with the positive will of our husbands. But I know a pregnant woman who wanted to come and stay here, but she couldn’t as her husband didn’t allow her to do so. Many men restrict women from leaving home for long periods (MWH user, FGD2).

#### Perceived high cost of MWH services

Some mothers who participated in the FGDs expressed reluctance to pay if the MWH service was linked to a payment. They said they would not be able to afford the cost of care provided at the MWH:

I don’t agree with payment for this service. … , I don’t know if they (women) could afford it, … (MWH non-user, FGD 1).

#### Misconceptions that limit access to the MWH – false information

Some participants thought that the MWH belonged to catholic nuns, while others thought mothers would be denied admission for lack of money. Some participants did not know that MWH services are covered by their community-based health insurance. In addition, they did not know that care for the most vulnerable pregnant women who are deemed poor according to Rwanda’s wealth categories, *Ubudehe* category 1 and 2; victims of gender-based violence; and teenage pregnancies are covered by the district administration social affairs office.

She might tell the nurse or the doctor about symptoms that do not seem serious because she is afraid of being admitted to the MWH. She might just share a few and yet she has a potentially serious condition *…* (MWH user, FGD 1).

### Supply-related barriers

#### Fear to operate at full capacity – ‘what if we admit all these women?’

At any given time, the hospital ensures that there is space in the MWH to admit more severe cases when they come. Additionally, the MWH does not have enough staff to provide adequate support to the women in the MWH and the hospital keeps rotating staff from time to time.

We ask ourselves many questions like – will we have enough staff to take care of them? What if we admit a certain woman and tomorrow, we get a more serious case, what would we do?’ So, it is a matter of balancing all the above (Ruli Hospital, KII 7).

#### Uncertainty over funding for MWH

All health providers reported that the MWH still receives some support from *Matris Mundi*, the founding organisation, as well as from some private funders. They noted that some funders are retired and are no longer sending enough support:

… it would be better if the hospital starts getting ready to take over [the responsibility of running the MWH]. (Ruli Hospital, KII 10).

#### Leveraging community meetings to improve awareness of MWH services

Most health providers suggested that messages to increase utilisation of the MWH be integrated into the existing home-grown community platforms, such as ‘*umuganda’* (a community gathering where residents meet once a month to conduct community work) and ‘*umugoroba w’ababyeyi’* (a forum for parents in the same village to come together in the evenings to discuss matters regarding the health and social issues affecting their families). In addition, respondents stressed the importance of working in partnership with community health workers and influential opinion leaders to reach the relevant audience.

There are community gatherings for various events […], … it will be a good time to share information about MWHs. This information can be delivered by CHWs who are trusted […]; this would increase awareness and encourage mothers to stay at the MWH (Ruli Hospital KII 1).

#### User fees as a barrier to utilisation of MWH

The MWH offers services at no extra cost to mothers. However, some key informants noted that cost is a barrier to the utilisation of the MWH due to women’s fear and misconception that MWH services come with additional costs.

*…* No one has ever been denied health care services because they have no money. They are just afraid. ‘[…] and don’t know that you pay the same fees as someone who is admitted in maternity (Ruli Hospital, KII 3).

## Discussion

Our study found that facilitators to the use of the MWH included perceptions that the MWH offers a peaceful environment and good-quality services, offered timely obstetric care services, and was associated with good outcomes while the barriers to the use of the MWH included limited awareness, fear of operating at full capacity, influence from partners, fear of cost, and misconceptions.

Most women noted that they had ‘peace of mind’ due to the good quality of obstetric care and psychosocial support they received. This is similar to findings from a study in Ethiopia where integration of health services and perceived high-quality comprehensive EmONC was a key facilitator for MWH utilisation [19].

Most women reported that they share their experiences with their peers when they return to their communities, which may further facilitate the use of the MWH as seen in another study in Ethiopia where positive word-of-mouth contributed to increased use of MWHs [19]. Additionally, MWH users were happy with the quality of services offered at the MWH and the good pregnancy outcomes. Similar findings were reported in studies from Ethiopia [18], Liberia [24], and Guatemala [20].

Similar to MWHs in Guatemala, the Ruli MWH provides women with culturally appropriate care. Women participated in routine activities such as cooking, sewing, making handicrafts, and other chores. They were allowed to bring food at will, though the food was provided at the MWH. The MWH was clean, and health providers visited the women regularly to assess their progress and shared health information regarding nutrition during pregnancy and breastfeeding, what to expect during delivery, and how to care for their babies, among others.

The MWH in Rwanda provides similar services as other MWHs in low- and middle-income countries, e.g. food, toilet and showers, and kitchen facilities [16]. In Ethiopia, utilisation of MWH was associated with the availability of food at the MWH, and the freedom to practice their cultural ceremonies such as cooking [18], which is similar to our findings. These activities create a homely environment which may contribute to increased utilisation.

In terms of positive outcomes, MWH users and healthcare providers pointed out that ‘most of the women who come here go back home with their babies’ and that no pregnant woman has died while giving birth among those who used the MWH. This finding is supported by quantitative studies which reported that MWH users had better outcomes, including zero maternal death, fewer deliveries by caesarean sections, fewer cases of postpartum haemorrhage, and reduced stillbirths [15].

Our study noted that women were happy with the timely maternity care that was provided before and after the onset of labour. Health providers at the MWH assess progress and provide immediate obstetric care when labour begins. This is backed up by findings from our previous study where MWH users had fewer complications such as postpartum haemorrhage, birth asphyxia, and stillbirth, compared to non-users [15].

In Rwanda, women are homemakers and take care of their families. Absence from home necessitates finding someone to take care of the children and/or husband. Our study found that women had to consult their partners before using the MWH and were worried about who would take care of their husbands and children while at the MWH. Some women depended on their mothers-in-law to decide for them to stay at the MWH. This lack of autonomy in such decision-making was also noted as a barrier to MWH use in Zambia [21]. Besides, some women felt the need to have a companion present with them at the MWH, which is not allowed at the MWH.

Studies in SSA have identified cost as a barrier to the use of MWHs [21,25,26]. In Zambia, for example, women paid for food (65%) and user fees (50%) during their stay at the MWHs, also contributing to the loss of earned income (35%) [27]. However, our study found that most non-users perceived the costs of services at the MWH as a barrier despite the services being offered at no additional cost, yet services at the MWH are covered by health insurance.

The MWH is managed by Ruli Hospital in partnership with a private funder. Currently, the MWH is run by a social worker who also works as a cleaner and is supported by nurses and midwives from the hospital to provide routine obstetric care. Some participants expressed concern that the MWH may not be sustained in the future due to reduced financial support from donors. Similar concerns were raised in studies in Zambia [28] and Guatemala [20], impacting the sustainability of the MWH. Our study noted that mothers were reluctant to pay for MWH if they were required to pay. To ensure that the MWH is sustainable, various resource mobilisation efforts should be explored including initiating income-generating activities, donations from the community, and increased budgetary allocation from the hospital.

Our study noted limited awareness of the existence, purpose, and services at the MWH as a reason for not using the MWH, which was also identified as a barrier in other studies in central Ethiopia [29]. The MWH has not been involved in community mobilisation, and most referrals to the MWH come from health centres and the hospital’s antenatal care clinic. Moreover, our previous study found that a large proportion of women with indications for admission to the MWH did not use it [15], which indicated a probable gap in awareness about the existence of the MWH. Participants recommended raising awareness of MWHs through existing community platforms and community health workers to address information gaps and dispel misconceptions that limit the use of MWH. Besides, the adoption of MWHs by the government as one of the maternal and child health interventions could help increase the visibility, awareness, and use of MWHs in general.

The MWH has a 15-bed capacity, limiting the number of women who can be admitted at a given time. The MWH applies certain admission criteria which may exclude some women, to reserve space for women who meet the criteria. Bed occupancy varies from time to time, and it is very hard to predict when the MWH will be full or not. Similar findings were observed in another study in Zambia where bed occupancy varied between 13% and 151% and planning for MWH capacity was very difficult [30]. Therefore, the MWH would need additional beds so that health providers can be more confident in admitting more women.

Preliminary evidence from the only existing MWH at Ruli Hospital in Rwanda shows the potential for the MWH to contribute to improving maternal and child perinatal outcomes [15]. Considering the stagnating maternal mortality ratio [4], Rwanda could scale up MWHs to boost the existing interventions and improve maternal health outcomes.

Successful implementation of MWH is dependent on its integration into the health system to allow for continuous care [31]. During integration, facilitators, barriers, and other lessons learnt from the Ruli MWH experience should be considered to define a package of MWH services for Rwanda.

## Strengths and limitations

The concept of MWH is not widely known in Rwanda, and so is its documentation. Consequently, there is a lack of local literature to compare with or give context to our findings. Our study was conducted in one MWH since it was the only one in the country. To make up for this limitation, we recruited women without considering their place of residence to ensure representation of other districts. In addition, we established trustworthiness of the study using various strategies [32]. First, we collected data from stakeholders who were aware of, or had interacted with, the programme. Secondly, we audio-recorded all FGDs and KIIs for verification. Thirdly, we ensured credibility through triangulation using FGDs and KIIs, diverse groups of participants coming from various locations within and outside the district, and the first three authors analysed the data. To avoid researcher bias, the first three authors independently read and re-read the transcripts and coded them line by line to identify meaning units. Lastly, the research team conducted debriefing sessions, promoted peer scrutiny of data collection and analysis, and used thick descriptions in explaining identified themes. The researchers also described the context to allow for the transferability of the findings.

## Conclusion

This is the first study of its kind in Rwanda and highlights key factors for consideration to improve the only existing MWH and possibly scale up the intervention to other districts. The MWH offers a peaceful environment for pregnant women while providing quality and timely obstetric care resulting in positive outcomes for women who stay at the MWH. However, the existence and benefits of the MWH are not widely known in the community, and its use is limited due to inadequate resources as well as the perceived high cost of MWH services. There is a need for increased awareness of the MWH among healthcare providers and the community and potential piloting of MWH in other districts to inform the adoption of the MWHs in Rwanda, which is critical in addressing the needs of high-risk pregnant women in rural Rwanda.

## Paper context

The World Health Organization recommends the implementation of MWH to reduce delays in access to obstetric care for high-risk pregnancies. This recommendation has not been implemented in Rwanda, and there is a gap in knowledge regarding MWHs. Using data collected from the only MWH in the country, we explored health provider and beneficiary perspectives on facilitators and barriers to the use of MWH. Our findings provide critical information for the scale-up of MWHs.

## References

[1] Lawn JE, Blencowe H, Oza S, You D, Lee AC, Waiswa P, et al. Every newborn: progress, priorities, and potential beyond survival. Lancet. 2014;384:189–8. 24853593 10.1016/S0140-6736(14)60496-7

[2] United Nations Children’s Fund. Levels & trends in child mortality: report 2012: estimates/developed by the UN Inter-agency group for child mortality estimation. 2012.

[3] World Health Organization. Trends in maternal mortality 2000 to 2017: estimates by WHO, UNICEF, UNFPA, World Bank Group and the United Nations Population Division. 2019.

[4] National Institute of Statistics of Rwanda (NISR), Ministry of Health, ICF. Rwanda demographic and health survey 2019-20. Kigali, Rwanda and Rockville, Maryland, USA: NISR, MoH and ICF; 2021.

[5] Benimana C, Small M, Rulisa S. Preventability of maternal near miss and mortality in Rwanda: a case series from the University Teaching Hospital of Kigali (CHUK). PLoS ONE. 2018;13:e0195711. 29944664 10.1371/journal.pone.0195711 PMC6019403

[6] Hategeka C, Arsenault C, Kruk ME. Temporal trends in coverage, quality and equity of maternal and child health services in Rwanda, 2000-2015. BMJ Glob Health. 2020 Nov;5:e002768. 10.1136/bmjgh-2020-002768 PMC7668303 33187962

[7] Rulisa S, Ntihinyurwa P, Ntirushwa D, Wong A, Olufolabi A. Causes of maternal mortality in Rwanda, 2017–2019. Obstet Gynecol. 2021 Oct 1;138:552–556. 10.1097/AOG.0000000000004534 34623066

[8] Ministry of Health Rwanda. Fourth health sector strategic plan July 2018–June 2024. Kigali: Ministry of Health; 2018.

[9] Dadi TL, Bekele BB, Kasaye HK, Nigussie T. Role of maternity waiting homes in the reduction of maternal death and stillbirth in developing countries and its contribution for maternal death reduction in Ethiopia: a systematic review and meta-analysis. BMC Health Serv Res. 2018;18:1–10. 30285757 10.1186/s12913-018-3559-y PMC6167854

[10] World Health Organization. Maternity waiting homes: a review of experiences. 1996.

[11] van Lonkhuijzen L, Stekelenburg J, van Roosmalen J. 2012. Maternity waiting facilities for improving maternal and neonatal outcome in low‐resource countries. Cochrane Database Syst Rev. DOI:10.1002/14651858.CD006759.pub3 PMC4098659 23076927

[12] McRae DN, Bergen N, Portela AG, Muhajarine N. A systematic review and meta-analysis of the effectiveness of maternity waiting homes in low- and middle-income countries. Health Policy Plann. 2021;36:1215–1235. 10.1093/heapol/czab010 34179952

[13] Bekele BB, Dadi TL, Tesfaye T. The significant association between maternity waiting homes utilization and perinatal mortality in Africa: systematic review and meta-analysis. BMC Res Notes. 2019;12:1–6. 30642355 10.1186/s13104-019-4056-z PMC6332606

[14] Scott NA, Kaiser JL, Ngoma T, McGlasson KL, Henry EG, Munro-Kramer ML, et al. If we build it, will they come? Results of a quasi-experimental study assessing the impact of maternity waiting homes on facility-based childbirth and maternity care in Zambia. BMJ Global Health. 2021;6:e006385. 10.1136/bmjgh-2021-006385 PMC8655557 34876457

[15] Tayebwa E, Kalisa R, Ndibaza AF, Cornelissen L, Teeselink EK, Kim Y-M, et al. Maternal and perinatal outcomes among maternity waiting home users and non-users in Rural Rwanda. Int J Environ Res Public Health. 2021;18:11211. 34769730 10.3390/ijerph182111211 PMC8583170

[16] Smith S, Henrikson H, Thapa R, Tamang S, Rajbhandari R. Maternity waiting home interventions as a strategy for improving birth outcomes: a scoping review and meta-analysis. Ann Glob Health. 2022;88. DOI:10.5334/aogh.3496 PMC8782095 35087708

[17] Ethiopian Public Health Institute. Ethiopian Emergency Obstetric and Newborn Care (EmONC) assessment 2016 final report. Addis Ababa: Ethiopian Public Health Institute, Federal Ministry of Health. 2017;478.

[18] Selbana DW, Derese M, Sewmehone Endalew E, Gashaw BT. A culturally sensitive and supportive maternity care service increases the uptake of maternity waiting homes in Ethiopia. Int J Women’s Health. 2020;12:813–821. 33116931 10.2147/IJWH.S268245 PMC7553138

[19] Vermeiden T, Schiffer R, Langhorst J, Klappe N, Asera W, Getnet G, et al. Facilitators for maternity waiting home utilisation at Attat Hospital: a mixed‐methods study based on 45 years of experience. Trop Med Int Health. 2018;23:1332–1341. 30286267 10.1111/tmi.13158

[20] Ruiz MJ, van Dijk MG, Berdichevsky K, Munguía A, Burks C, García SG. Barriers to the use of maternity waiting homes in indigenous regions of Guatemala: a study of users’ and community members’ perceptions. Culture Health Sexuality. 2013;15:205–218. 23234509 10.1080/13691058.2012.751128

[21] Sialubanje C, Massar K, van der Pijl MS, Kirch EM, Hamer DH, Ruiter RA. Improving access to skilled facility-based delivery services: women’s beliefs on facilitators and barriers to the utilisation of maternity waiting homes in rural Zambia. Reprod Health. 2015;12:1–13. 26148481 10.1186/s12978-015-0051-6 PMC4493824

[22] National Institute of Statistics of Rwanda (NISR). Ministry of Finance and Economic Planning (MINECOFIN) [Rwanda]. Fourth Population and Housing Census 2012: Census Atlas. 2015.

[23] Anderson CA, Bushman BJ, Bandura A, Braun V, Clarke V, Bussey K, et al. Using thematic analysis in psychology using thematic analysis in psychology. Psychiatr Q. 2014;887:37–41.

[24] Horton R, Lee H, Perosky JE, Kofa A, Lori JR. Comparison of quality, birth outcomes, and service utilization between health facilities with and without maternity waiting homes in Liberia. Midwifery. 2022;105:103235. 34959000 10.1016/j.midw.2021.103235 PMC8811480

[25] Andemichael G, Haile B, Kosia A, Mufunda J. Maternity waiting homes: a panacea for maternal/neonatal conundrums in Eritrea. J Eritrean Med Ass. 2009;4:18–21.

[26] Chibuye PS, Bazant ES, Wallon M, Rao N, Fruhauf T. Experiences with and expectations of maternity waiting homes in Luapula Province, Zambia: a mixed–methods, cross-sectional study with women, community groups and stakeholders. BMC Pregnancy Childbirth. 2018;18:1–10. 29370773 10.1186/s12884-017-1649-1 PMC5785796

[27] Lee H, Maffioli EM, Veliz PT, Sakala I, Chiboola NM, Lori JR. Direct and opportunity costs related to utilizing maternity waiting homes in rural Zambia. Midwifery. 2022;105:103211. 34894428 10.1016/j.midw.2021.103211 PMC8811481

[28] Buser JM, Moyo B. Agness mseteka: maternity waiting home caretaker and protector of pregnant women in Rural Zambia. Ann Glob Health. 2021;87. DOI:10.5334/aogh.3253 PMC8231459 34221907

[29] Dereje S, Yenus H, Amare G, Amare T. Maternity waiting homes utilization and associated factors among childbearing women in rural settings of finfinnee special zone, central Ethiopia: a community based cross-sectional study. PLoS ONE. 2022;17:e0265182. 35298504 10.1371/journal.pone.0265182 PMC8929623

[30] Vian T, Kaiser JL, Ngoma T, Juntunen A, Mataka KK, Bwalya M, et al. Planning for maternity waiting home bed capacity: lessons from Rural Zambia. Ann Glob Health. 2022;88. 10.5334/aogh.3691 PMC9138814 35651969

[31] Penn-Kekana L, Pereira S, Hussein J, Bontogon H, Chersich M, Munjanja S, et al. Understanding the implementation of maternity waiting homes in low- and middle-income countries: a qualitative thematic synthesis. BMC Pregnancy Childbirth. 2017;17:1–12. 28854880 10.1186/s12884-017-1444-z PMC5577673

[32] Korstjens I, Moser A. Series: practical guidance to qualitative research. Part 4: trustworthiness and publishing. Eur J Gener Pract. 2018;24:120–124. 10.1080/13814788.2017.1375092 PMC8816392 29202616

